# The changing self: The impact of dementia on the personal and social identity of women (findings from the Improving the Experience of Dementia and Enhancing Active Life programme)

**DOI:** 10.1177/14713012211047351

**Published:** 2021-10-12

**Authors:** Hannah Scott

**Affiliations:** School of Clinical Medicine, 2152 University of Cambridge, England

**Keywords:** dementia, self, selfhood, self-esteem, women

## Abstract

This paper explores the impact of dementia on the selfhood of women, specifically the ways in which changes occur as a result of such a diagnosis. Interviews were conducted with 12 women (recruited from the Improving the Experience of Dementia and Enhancing Active Life programme dataset), and analysed using interpretative phenomenological analysis. Emergent themes concerned the process of receiving and adjusting to a dementia diagnosis, the emotional and psychological impact of dementia, self-presentation and stigma and the self-enforcement of new boundaries. The analysis showed that dementia had a wide-ranging impact on the selfhood and identity of women, with newfound characteristics associated with the disease leading to a loss of self-esteem, sadness and anger. The women subsequently engaged in the modification of their behaviour, as a means of coming to terms with the losses experienced.

## Introduction

Women have been described as a ‘marginalised majority’ within dementia research (Alzheimer’s Research UK, 2015, p. 1). Their subjective experiences have remained largely neglected, despite women making up 61% of the population diagnosed with the disease, and dementia being the leading cause of death for women in the United Kingdom. Savitch et al. (2015) stressed the need to encourage women’s voices in the dementia literature, and to consider the diversity of roles that constituted selfhood – including occupational histories, as well as familial roles.

The concept of selfhood in dementia research has been based on different models and approaches (Caddell & Clare, 2010). Studies have presented selfhood as being equated with memory and language (Capps, 2008), or autobiographical memories (Addis & Tippett, 2004), whilst others have explored selfhood through the accounts of caregivers (Ward, 2015). Such studies have concluded that selfhood is lost in dementia. However, those studies that have adopted an exploratory approach to selfhood, allowing people with dementia to construct personal narratives, have presented a far more nuanced picture (e.g. Buse & Twigg, 2015; Tatzer, 2019).

Selfhood was conceptualised in this study, according to the social constructionist approach outlined by Sabat and Harré (1992). This purports that selfhood can be explored through the construction and co-construction of narratives, and puts the person with dementia at the forefront of research (Sabat, 2001). It takes into account how both personal and social identity are impacted by the disease; that is to say, how the person with dementia perceives themselves, and how this has changed, as well as how social networks and relationships are constructed within narratives. Self-esteem – the evaluative–affective appraisal of one’s characteristics (Garaigordobil et al., 2008) – is a key component of the social constructionist model, and qualitative methods, such as interviews, are an appropriate tool for exploring why someone’s self-esteem may have changed as a result of a diagnosis of dementia.

Self-esteem among people living with dementia was explored using a social constructionist case study approach by Sabat et al. (1999). Since then, whilst self-esteem has not been widely discussed in the dementia literature, and is particularly limited in the qualitative domain, research into chronic illness, more generally, has found that there is a threat to self-esteem, for people living with breast cancer (Drageset et al., 2018), Parkinson’s disease (Haahr et al., 2011) and Chronic Obstructive Pulmonary Disease (Nicolson & Anderson, 2003). Cheston et al. (2015) discussed self-esteem and social connectedness in the context of dementia, and found that investing in the things that gave them a sense of worth, was important for people with dementia, as was the ability of those around them to foster a caring social environment that supported the person to forge meaningful connections.

Relatedly, stigma is an important concept for the present study because it can lead to a person becoming ‘discredited’ and thus reflect back on how they perceive themselves (Goffman, 1963, p. 4). Research has shown that dementia symptoms can be deeply stigmatising, with other people avoiding social interaction with the individual in question (Graham et al., 2003). Moreover, the acquisition of such a label then results in these cultural stereotypes becoming personally relevant (Garand et al., 2009). Both felt stigma and enacted stigma are relevant to this study. The former refers to the shame a person feels about having a particular condition – an internalisation of public stigmatic attitudes (Corrigan & Watson, 2007). The latter refers to experiences of discrimination (Scambler, 1989). Stigma is especially important, given that women face a ‘triple jeopardy’ of age, gender and dementia-based discrimination (Bamford & Walker, 2012, p. 123). In exploring exposure to negative stereotyping around ageing and dementia, Scholl and Sabat (2008) concluded that this could have a profound impact once a person was then diagnosed with the disease in later life, leading to self-stereotyping.

This paper draws on data from a doctoral study that sought to explore the impact of dementia on the selfhood and identity of women. The study was funded by the Economic and Social Research Council as part of the Improving the Experience of Dementia and Enhancing Active Life (IDEAL) programme of research (Clare et al., 2014). IDEAL is a large, UK-wide cohort study exploring what it means to live well with dementia. The paper focuses on the notion of change, the ways in which the women believed they had changed as a person, the ways in which their self-esteem had been affected by dementia and the changes in their behaviour as a result – for example, in terms of self-presentation and managing new boundaries. It takes the view outlined by Sabat and Harré (1992) in their theory of selfhood and dementia, that self-esteem is embedded within the appraisal of one’s attributes and characteristics, and is also a product of the social environment, which can mediate self-esteem. Thus, qualitative methods offer an ideal opportunity for exploring issues related to the above. The study gave primacy to the lived experiences of women, who have been underrepresented in the dementia literature. It builds on previous research, exploring how the loss of past attributes and roles impacts on the self-esteem of women, whilst also considering the significance of occupational identities, which are usually associated with the selfhood of men. The study includes a larger, more diverse sample of women than much of the existing literature (e.g. Borley & Hardy, 2017; Robertson, 2014), which will help to shed light on how women from different social backgrounds and with different life experiences are impacted by dementia.

## Method

### Design

The methodological approach underpinning this study is Interpretative Phenomenological Analysis (IPA) (Smith et al., 2009). IPA is focused on the experiential; how people make sense of and talk about their life experiences. This makes it a particularly suitable method for exploring the impact of a major life event, such as a chronic illness. Theoretically, IPA is founded on three key principles: phenomenology, hermeneutics and idiography. It is phenomenological, in that it is committed to the subjectivity of individual experience. Since IPA involves the researcher making sense of – or interpreting – the participant making sense of these experiences, it is also hermeneutic (Smith, 2004). Finally, IPA is idiographic, in that it assumes an individual focus, and explores how a person interprets his or her world at any given point. The ‘finely-textured analysis’ with which IPA is concerned, assumes that a person can offer a unique perspective on a particular phenomenon (Shinebourne, 2011, p. 22).

### Participants

Twelve women with a diagnosis of Alzheimer’s disease and/or vascular dementia were recruited to the study from the main IDEAL cohort (see Table 1). IDEAL is made up of 29 National Health Service memory clinics, working in partnership with the research study team. The sample was drawn from three sites (two in England and one in Wales). All men were excluded from the study, as were women with an Mini-Mental State Exam score of 20 or less, which would indicate moderate or severe dementia (Department of Health, 2014). Those participants who met the inclusion criteria were sent invitation letters, specifying that this project fed into the IDEAL study, in which they were already taking part, as well as a short summary of the project in lay language. The letters were followed up with a phone call, where more detail was provided, and participants could ask any questions. A date for the interview was then set up, where participants were talked through a study information sheet and asked to complete a consent form. Thus, consent was obtained both verbally and in writing. The participants came from a range of social backgrounds. Some lived alone, whilst others lived with a partner or spouse. In this paper, their names have been replaced with pseudonyms to protect anonymity.

**Table 1.** Participant characteristics.

Participant pseudonym	Age range	Previous occupation
Alice	70–74	Nurse
Betty	70–74	Nursery nurse
Bridget	70–74	Lorry driver
Catherine	75–79	Hairdresser
Flora	75–79	Bus conductress/post-lady
Joan	70–74	Secretary
Lynda	65–69	Civil servant
Marian	85+	Psychologist
Martha	80–84	Keyboard operator
Sally	70–74	Hairdresser
Stella	70–74	Hairdresser
Wendy	70–74	Teacher

The IDEAL study was approved by the Wales Research Ethics Committee 5 (reference 13/WA/0405), and the Ethics Committee of the School of Psychology, Bangor University (reference 2014–11684). The present study was approved as a substantial amendment to the above, and letters of access were obtained from each individual research site used. The IDEAL study was registered with UKCRN, registration number: 16593.

### Data collection

Once consent had been obtained, audio-recorded, semi-structured interviews were conducted with the women. These lasted between 40 min and 2 h. The interview guides contained deliberately open-ended questions, and were designed in such a way as to allow participants’ experiences to come to the fore. They addressed different areas of the participants’ lives, including their upbringing, their experience of being diagnosed with and living with dementia and their interpersonal relationships and social networks.

### Data analysis

The interview transcripts were analysed according to the step-by-step IPA method laid out by Smith et al. (2009), with some adjustments. Initially, each transcript was read and re-read several times, and notes made in the margins of the pages. Next, the content and language used by the participants was explored in more depth, and a detailed commentary written, that was broken down into three distinct categories. Descriptive comments used the researcher’s own words to interpret what the participant had said. Linguistic comments focused on aspects of language and language use. Conceptual comments engaged with the text at a more interrogative level – this final category signifying a shift towards the researcher embarking on the journey of interpretation, fundamental to the process of IPA (Smith et al., 2009). The third stage of the analytic process involved the drawing out of themes from the data, using the researcher’s notes as the basis for these. The themes identified from each transcript were listed, and connections sought between them – a process Smith and Osborn (2015, p. 43) refer to as ‘clustering’. Those themes that did not fit into a particular cluster were discarded. The final groups of themes were reviewed, and a superordinate label given to each. It is important to note that recurrence of themes was considered important; presence in over half the cases would define a superordinate theme. Sub-themes, however, did not have such a requirement. Sub-themes were included, even if they were reflected in the accounts of only a few participants. This ensured that the importance of individual experience was captured.

## Findings

This paper addresses the first superordinate theme to emerge from the data: *The Changing Self*. This theme also contains three sub-themes (see Table 2).

**Table 2.** Overarching theme and sub-themes.

The changing self
Receiving and adjusting to a diagnosis of dementia
The emotional and psychological impact of dementia
Self-presentation and stigma
Boundary maintenance

### Receiving and adjusting to a diagnosis of dementia

The women in this study had differing reactions to receiving a diagnosis of dementia. This was linked to their representations of what dementia actually was, what it constituted, in terms of being an illness, or part of the normal process of ageing. It was also linked to their experience prior to receiving a diagnosis. In both Betty and Lynda’s case, close family members were dismissive of their insistence that something was wrong. Hence, receiving a formal diagnosis constituted a sense of relief and vindication for these two women.

*She’s saying: ‘No, you’re just getting older, Mum’. And I’m saying: ‘No, it’s not that. This is different’. I don’t want to be like this, and I want to know what it is. And that’s why I was pleased when they gave me a diagnosis*. (Betty)

*It was three years it took me to be diagnosed. That…when she told me, it was the relief, you know? Because nobody was believing me. And even Joe started calling me a hypochondriac*. (Lynda)

Other participants experienced a powerful, negative reaction to being diagnosed with dementia. The accounts of Sally and Bridget laid bare the life-changing impact associated with this. Bridget, in particular, consistently aligned the disease with serious mental illness, of the kind that would previously have seen people sectioned in an asylum.

*I knew I had problems. I knew I wasn’t just forgetting things…that it was something more. They did a scan and they told me on Black Friday, would you believe? What a day. I’ll never forget that. Worst day of my life.* (Sally)

*I thought to myself, that’s it. I’m signed to the bin. All sorts of things were going through my mind.* (Bridget)

Still other participants expressed mild confusion or surprise at their diagnosis. This could be indicative of the fact that, amongst the general public and indeed, in the realm of science, relatively little is known about dementia. Therefore, the lack of clarity in the accounts of these women could reflect the uncertainty of about it meant for their lives.

*I was quite surprised. I thought, no, they’ve got it wrong [laughs]. But I mean…dementia…I don’t really know what it means, to be honest with you. I suppose it’s something that comes when you’re between seventy and eighty.* (Catherine)

*Well, the word ‘dementia’. I don’t really…perhaps I have come across it before, but not really knowingly. And I think it was a bit of a shock when it’s got a name to it. I didn’t feel that I came under that category.* (Joan)

This sub-theme has illustrated the different reactions that women have to being diagnosed with dementia, based on their conceptualisation of the disease. Some equated dementia with a pathological condition, others as part of the normal ageing process. Still others expressed uncertainty about what it meant. These differing understandings are important because they can mediate the emotional and psychological responses of women to the disease.

### The emotional and psychological impact of dementia

Many of the women in this study struggled with feelings of depression, anxiety and loneliness. Their self-esteem was impacted by the diagnosis of dementia, and led participants to negatively compare their present attributes with their former attributes. Sally talked about feeling that she was a different person now – on a fundamental level, she had changed as a result of dementia. She wondered aloud why her partner would put up with such changes, and concluded that she was powerless to stop it.

*That’s the biggest thing I’ve noticed – my personality. I’m not the Sally I used to be. I know I’m different. And he definitely knows I’m not the Sally I used to be. Makes me wonder sometimes how on earth he puts up with me. I know I’m different. That does bother me. Particularly when I’m nasty to Howard. I think, why does he put up with it? Like he says: Well, I love you’. But I don’t know why he puts up with it. That’s the sad bit, I think. I’m not the person I was. And that…bothers me. But there’s nothing I can do about it.* (Sally)

Some women described the losses that had taken place, in terms of role identities. Lynda, for example, had cared for her learning-disabled daughter, until her dementia diagnosis, and was the person others had relied upon. This role within her family had been central to how Lynda had perceived herself. Now, she described stark discrepancies between the past and present, the loss of such a valued set of attributes and responsibilities leading to damaged self-esteem.

*I was always the one in charge. I was really strong. Strong-minded and…you know. I was the boss in the house. If anything needed doing, I was the one that’d do it. And because my daughter’s got learning disabilities, I was the one that dealt with all the benefits and anything else that needed doing. If she’d got bills to pay, I used to help her. And now…well, she doesn’t rely on me for anymore, for anything. I’m the one that relies on everybody else. So compared to what I used to be like, I’ve changed ever such a lot. I just haven’t got the confidence anymore. I can’t rely on myself anymore.* (Lynda)

Other participants expressed a similar sense of their self-esteem being impacted by dementia. Betty, for example, also made direct comparisons with the past. She had previously described herself as ‘brash’ and ‘confident’. Now, she was more uncertain, experiencing self-doubt in social interactions and expressing concern over how other people perceived her.

*I was always quite a positive person, but now I think more about how other people perceive me than I would ever have done. Once I was brash, but I felt confident enough that I was doing what was right for me. I still do think, what’s right for me, but I question it more now, as to whether people will see the same thing of me as I see.* (Betty)

Some participants, having experienced a loss of self-esteem, withdrew from social life because of a perceived inability to cope with social situations. As a consequence, these women experienced loneliness.

*It has made me feel isolated in a way, because I haven’t got anything to talk about. And the other thing is, because I do try and act normal, but I don’t feel that I am.* (Lynda)

*I just feel lonely, I suppose – permanently. I feel as though I’m on my own, and I’m fighting my own corner.* (Bridget)

This sub-theme highlighted the wide range of emotional and psychological responses to living with dementia, expressed by women in the present study, including sadness and grief. Participants’ concerns spanned both social and functional areas, including in some cases, the altering of the fundamental perception of oneself. The notion of change was further highlighted in the ways that women made negative comparisons with aspects of the past and previously established identities. These changes to selfhood influenced, and were influenced by, stigma and the ways in which the women presented themselves in social situations.

### Self-presentation and stigma

Almost every participant in the present study was engaged in some form of self-management, that is to say, they were actively considering when, and to whom to disclose their illness. Many of the women chose to conceal the fact that they had dementia, for a variety of reasons, including fear of burdening their family members.

*I put on an act. I find it’s real hard work, but that’s what I’ll do most of the time, round my partner and round everyone. I don’t want them to worry.* (Lynda)

Whilst Lynda concealed symptoms of her illness so as not to cause her family worry and stress, Bridget had experienced negative reactions from other people, which had resulted in her changing her behaviour. Therefore, having previously disclosed the fact that she had dementia, Bridget now concealed this, to reduce the risk of being stigmatised further.

*In the beginning – maybe I shouldn’t have, but: ‘Well, I’m sorry, I can’t do that because I’ve got dementia’. And they’re like: ‘Uh, uh…’ So I panicked. I thought people would understand in this day and age.* (Bridget)

For some women, the idea of disclosing their illness was unthinkable. Catherine, for example, believed that doing so would make the other person uncomfortable. To her, this had the potential for social embarrassment. Catherine was also extremely conscious of what she considered to be ‘bad manners’ – which included forgetfulness. Thus, she relied heavily on her diary as face-saving mechanism and to conceal such symptoms.

*I don’t say: ‘Forgive me, I’ve got dementia’. I would think that would make them feel rather awkward. You know…yes, probably I am bluffing my way through things.* (Catherine)

Stella was another participant who chose not to disclose her symptoms to people outside of her immediate family. Stella experienced self-stigma – she considered dementia to be stigmatic condition – and thus expected to be stigmatised by others.

*If I said to you: ‘Ooh yes, I’ve got dementia’, then it’d be like that [recoils] wouldn’t it? And everyone…they don’t seem to know any different.* (Stella)

In a similar vein to Stella, Martha had not disclosed her illness to anyone but close family members. Her reasoning for this was slightly different, however. Martha did not appear to feel that she had acquired a stigmatic identity as a result of dementia, and instead asserted that her continued ability to do the things she always had, meant that she did not have the disease – which she associated with a high level of incapacity.

*None of my friends know that I’ve got dementia. I don’t feel as though I’ve got it myself to be honest with you. And I wouldn’t say that I’ve got dementia. I don’t tell people: ‘Oh I’ve got dementia’. Cause I just live my life like I’ve always lived it. Yeah…I never discuss it with anybody.* (Martha)

Unlike other participants, Betty opted to disclose her illness. It was important to her that people knew there was a reason why she could not find a word; she did not want to be thought of as stupid for having memory lapses, or rude, for interrupting a conversation. Betty was also heavily involved in advocacy work, and repeatedly expressed a desire to make dementia more visible. Hence, another reason why she disclosed her illness was to reduce the stigma associated with the disease.

*I want people to be aware that if I can’t find a word, there’s a reason why I can’t find that word. Or if my memory slips…or I might jump to something else, unrelated, it’s only that I’ve thought of it and had to open my mouth whilst I remember it. But anyway, I want people to be aware that it’s happening more.* (Betty)

Many of the women in the present study were acutely aware of the stigma attached to dementia, either as a result of first-hand experiences or the anticipation of such experiences. Stigma posed a threat to the ability of participants to construct a valued social identity, whilst also reflecting back on how they perceived themselves. Thus, participants engaged in self-management strategies, as a means of protecting themselves from this threat. These strategies were also used to downplay the symptoms experienced, and prevent family members from worrying, such as in the case of Lynda. Self-management strategies and an active consideration of whom to disclose their illness to were part of a more wide-ranging reassessment of boundaries for the women in this study.

### Boundary maintenance

Another way in which changes to the lives of the women in this study had taken place was in relation to boundary maintenance. Participants’ confidence in their abilities had been impacted by dementia, to the point where previously taken-for-granted activities were revaluated. Driving was one such activity, and was central to the identities of several women, it having formed a part of valued occupational or familial role identities in the past. Flora, for example, had been the family’s sole driver, but she no longer drove long distances. However, by focussing on her retained capabilities, namely, the fact that she could still drive, and felt comfortable doing so, the impact of the modification of her driving habits on Flora’s selfhood was lessened.

*I mean I still drive, and I feel quite happy driving. But I wouldn’t drive a long distance.* (Flora)

Whilst the mere preservation of the ability to drive was enough for Flora, other women struggled to come to terms with their self-imposed restrictions. Bridget had been a lorry driver, and she talked at length about her enjoyment of this career. Now, however, there appeared to be a conflict between her evident desire to drive, and the self-doubt that served to moderate her behaviour. Bridget’s driving habits now consisted of a twice-weekly trip to a dementia support group, which did not mediate the sense of loss.

*I want to drive, but then I’m beginning to think…under normal circumstances, wouldn’t bat an eyelid. But with this, I’ve got to think about it. I’ve got to remember that I’m not normal and I can’t just zoom off. Whereas I did before and never batted an eyelid.* (Bridget)

Similarly, Lynda’s career had involved a significant amount of long-distance driving. Now, she found herself having to moderate the length of car journeys due to the tiredness from which she frequently suffered as a result of dementia. Unfortunately, the renegotiation of her boundaries with regards to driving meant that Lynda was forced to stop attending her weekly support group sessions.

*When I go, I can drive there and I can drive back. The problem is, if I drive there, after an hour, an hour and a half of talking, I then start getting quite tired, so I didn’t feel that I should drive back.* (Lynda)

Aside from driving, the women had made other changes to the ways in which they went about aspects of their lives. Bridget had stopped going for long walks in the countryside, after getting lost and having a panic attack. This singular event had a dramatic impact on her walking habits, although Bridget still made a conscious effort to continue with an activity that she had always loved, and that was important to her well-being.

*I can go through the gate and pick the paper up from the paper boy. See somebody, have a natter on the road and that…as long as I don’t go out of the area where I feel safe.* (Bridget)

Sally was another participant who had significantly restricted her activities. Having previously taken regular trips into town, now Sally was fearful of social interaction and being amongst crowds of people caused her stress and anxiety. Thus, the grounds of the assisted living community where she lived now represented the boundary of safety for Sally.

*I don’t feel right out there. I feel…more vulnerable out there. Not happy with people around me at all. I feel like everybody’s looking at me. And I know they’re not, but that’s just the way I feel.* (Sally)

This sub-theme has shown that the women in the present study engaged in the management and renegotiation of boundaries. They thus changed their behaviour as a result of dementia – or dementia had impacted on their confidence in their ability to do things in the same way as they had previously.

## Discussion

This paper illustrates the ways in which women living with dementia account for the changes they identify in their sense of self. The data show some of the ways in which the women in the present study perceive themselves as having changed. It explores, through four sub-themes, the impact of an initial diagnosis in terms of how dementia is differentially conceptualised, and the emotional reaction to the losses experienced to aspects of selfhood. It then considers how women engage in strategies of disclosure and non-disclosure, based on how they perceive themselves, and finally, the ways in which behaviour is modified, to adapt to the changes experienced.

The women expressed a variety of responses to being diagnosed with dementia, including relief, shock and confusion. This was dependent on how they conceptualised the disease, which supports the work of Clare et al. (2016) on the illness representations held by people with dementia. Thus, whilst some people conceptualised their diagnosis in terms of an illness or disease, others attributed it to the ageing process, or dismissed the notion of dementia entirely. Those whose representations of dementia aligned with the illness/disease view, appeared to have better prepared themselves for the diagnosis, and had greater awareness, but they were also more anxious, sad and expressed concern for future implications. For this latter group, there was a kind of ‘existential gravity’ associated with the diagnosis (Williams, 1984, p. 197). Moreover, it is possible that what on the surface could be interpreted as a lack of acceptance or understanding in the second group, could in fact serve a protective function, allowing the women to normalise their illness and carry on as before (Clare et al., 2016).

For many women, there was a struggle to adjust to a diagnosis of dementia, coupled with a recognition of the losses, which can lead to a range of emotional and psychological responses. These participants found it hard to reconcile their limitations, and had decreased self-esteem and confidence in their abilities. They drew negative comparisons with their former attributes, lamenting the ways in which these had changed or been lost. The ways in which these women perceived themselves had fundamentally changed; they experienced what Charmaz (1983, p. 168) described as a ‘crumbling away of their former self-images’. Aminzadeh et al. (2007) concluded that identity loss was one of the most feared losses in dementia. Sometimes this can be related to the social stigma of the disease, but not always. The changes in the power dynamics of role-based relationships can pose a significant threat to the person with dementia (Wadham et al., 2016). In this study, for example, there was a clear contrast between how Lynda presented her former and present self. Losing the ability to care for her daughter, her status as being ‘the one in charge’, and the person on whom others relied, which had been central to Lynda’s familial and gendered role identities, threatened her entire sense of self. Now, there was a shift whereby she had become reliant on other people, and her self-esteem was damaged as a result. This fits in with the work of Olsson et al. (2013), who found that losses perpetuate damage to self-esteem, which arises as a lack of trust in oneself and feelings of insecurity prevail.

A lack of self-confidence in one’s ability to navigate the normal realms of life can lead to loneliness and isolation. In his description of social anxiety, Bandura (1997) observed that the person may withdraw from social situations because they believe they do not have the capacity to meet normal social standards. It is important to note that this perception may not necessarily be reflective of reality, but either way, it fosters ‘defensive avoidance’ of certain activities that may expose the person to threats (Bandura, 1997, p. 319). Loneliness – the ‘discrepancy between desired and available relationships’ (Walton et al., 1991, p. 169) – then occurs as a result of this withdrawal. Weeks (1994) further makes a distinction between emotional and social loneliness. The former is described as the loss of intimate relationships, the latter as the loss of meaningful friendships. Most participants in the present study evidenced social loneliness, and a lack of belief in their ability to mobilise social support, which could have alleviated this (Smith et al., 2000). Evidence has suggested that people who have chronically low self-esteem will experience a greater decline in agentic capabilities with ageing – in other words, will withdraw from social interactions and experience an increased sense of loneliness (e.g. Fry, 2001; Smith et al., 2000). This did not hold true for the women in the present study, however. Those who described feeling lonely did so as a result of the threat posed to their self-esteem by dementia, and specifically compared their present situation negatively with the past, previously asserting that they had been confident, outgoing people.

As Beard (2004) observes, dementia is a non-visible condition (at least in the early stages), which affords the person a degree of choice in how they manage the awareness of symptoms within a social context. Almost all of the women in this study engaged in active consideration of whether to disclose their illness or not. These conscious choices about self-presentation served to lessen the perceived threat posed by social interaction (Clare, 2003), which in turn could protect self-esteem. The reasons given by the women for their choices often reflected their experiences within their social world, and the behaviour (intentional or not) of others around them (Kitwood, 1997). In contrast with other studies that found that most people with dementia would rather be open about their diagnosis (e.g. Beard, 2004), the women in this study largely attempted to conceal their symptoms. Some, such as Bridget and Stella, expected to encounter negative reactions from others, and thus had devised defensive non-disclosure strategies (Scambler, 1989). Lynda believed that being open about her symptoms or how she was feeling, would cause her family stress and worry. Catherine expressed a belief that it would be poor social etiquette to reveal her symptoms, and would cause people to feel awkward. Martha concealed her diagnosis because she played down its significance, reasoning that there was simply no need for people to know because it did not have an impact on her own life. Betty, in contrast, engaged in ‘protective disclosure’ (Charmaz, 1991, p. 119). In other words, she positioned herself as being in control and protected her sense of self by priming people that she had dementia, so as not to appear rude or stupid. Betty’s strategy of disclosure could also be considered a means of claiming active ‘social citizenship’ (O’Connor et al., 2018, p. 46). Betty wanted to be held up as a visible example of dementia, and to increase understanding of the disease. There has been little attention paid to the disclosure practices of the person with dementia to others within their social world. However, disclosure is linked to stigma, and consequently, to the ability to live well (O’Connor et al., 2018). Hence, it is important to explore such practices and what they mean for individuals living with dementia.

Awareness of the disease progression and the changes taking place can be observed in the way that women readjusted their boundaries. This was often linked to a lack of confidence and trust in their own abilities. Some women were able to renegotiate boundaries successfully, with minimal damage to their sense of self. Others found the process harder, particularly when the activity in question had been central to their identity and there was nothing to mitigate the narrowing of boundaries. Renegotiation could be observed in many accounts with reference to driving. Participants now only drove for shorter journeys, or only drove on particular days of the week, to a particular event. This reflects active consideration of the acceptable level of risk involved, when setting these renewed boundaries (Armstrong & Morris, 2010). Catherine and Flora were two examples of where renegotiation of boundaries was achieved successfully. The impact on these women was lessened, because they focused on their retained capabilities, namely, the fact that they were still able to drive – even if this had been modified. The mere preservation of this activity was enough. In contrast, Bridget and Lynda struggled with their self-imposed driving limitations. Both had considered themselves competent, trusted drivers, a belief that had now changed. This had given rise to a conflict between a desire to drive and perceiving themselves as constrained by their illness. Even for these women, however, the mere retention of the ability to drive was still important, described as ‘top of the tree’ by Bridget. Boundary maintenance was also evidenced with regards to walking. Bridget had changed her walking habits, but importantly, still made the effort to do shorter walks. Sally no longer ventured into town, rather stayed within her local area. There has been a significant amount of research carried out, that has explored how people with chronic conditions navigate boundaries. This has mainly focused on the restrictive impact of physical pain (e.g. Mackichan et al., 2013), but such findings can also be applied to the experiences of living with dementia.

This study has shown that dementia can have a wide-ranging impact on psychological, social, emotional and physical domains of the lives of women. Specifically, the women experienced significant changes to their self-concept and overall self-esteem. Previous research has suggested that where such damage occurs, it is as result of treatment by others. In other words, if people within the world of the person with dementia fail to cooperate in that person’s attempts to construct a valued social identity, this will reflect back on them, and selfhood will be impacted (e.g. Kelly, 2010; Sabat, 2002). That is to say, they will see themselves in accordance with how others see them. In the present study, this could be seen in the account of Bridget, for example, who had experienced negative reactions when telling people she had dementia, and which subsequently reflected back on how she saw herself.

Another way in which the literature has shown damage to selfhood to occur, is when it is described by people with dementia in terms of memory problems (Cadell & Clare, 2011). Yet, in such instances, people with dementia draw a distinction by insisting that the illness does not impact on how they see themselves; in other words, they are the same person as they have always been (Hedman et al., 2012), and there is an emphasis on the continuity with their pre-diagnosis selves (MacRae, 2010). For many women in the present study, however, this was not the case. These women experienced a more fundamental change to their sense of self, drawing comparisons between who they were now, and who they used to be. Moreover, this was not always linked to an inability to construct a valued social identity.

This study also sheds light on self-esteem as it relates to dementia. Previous literature that has explored self-esteem in people with dementia has done so largely by using quantitative measurements, such as the State Self–Esteem Scale (e.g. Cotter et al., 2018), or the Rosenberg Self-Esteem Scale (e.g. Lamont et al., 2019). Other literature has focused on the self-esteem of caregivers of people with dementia (Jütten et al., 2020). Exploring self-esteem through a qualitative lens allows for an understanding of why a person may have low self-esteem, as opposed to merely whether they have it or not. Many women in the present study experienced low self-esteem, borne out of decreased confidence in their abilities. They made comparisons with former attributes, highlighting the belief that they could no longer accomplish the things they had previously. To protect their sense of self-worth, the women avoided situations where they felt this was under threat. Relatedly, the women also engaged in active consideration of how they presented themselves and to whom they disclosed their illness, for fear of encountering stigmatic attitudes, which stemmed from a belief that they had acquired a stigmatic identity as a result of having been diagnosed with dementia.

### Implications of the study

Recognising the variability in people’s responses to dementia, and the reasons for it, is important in considering how people can be helped – for example, via counselling or other support services. This is similarly relevant for the significant others of people living with dementia, to ensure that they don’t view dementia in stereotypic or reductive terms. A ‘one-size fits all’ approach to helping people navigate their lives following a diagnosis of dementia does not comport with the facts of how women (and perhaps men as well) respond. Relatedly, this study emphasises the need for greater understanding of the emotional and psychological dimensions of dementia. There has historically been confusion associated with these, whereby they have been assumed to be a symptom of dementia, or where dementia has been misdiagnosed as depression (Scott et al., 2019). Instead, they should be acknowledged as a reaction to being diagnosed with the disease. Similarly, self-presentation strategies that suggest a lack of awareness on the part of the person with dementia could be construed as reactions to the person’s social environment and to instances of felt or enacted stigma.
